# Spatial and temporal patterns of malaria incidence in Mozambique

**DOI:** 10.1186/1475-2875-10-189

**Published:** 2011-07-13

**Authors:** Orlando P Zacarias, Mikael Andersson

**Affiliations:** 1 Department of Mathematics and Informatics (DMI), Eduardo Mondlane University, Maputo, Mozambique; 2 Department of Computer and System Sciences (DSV), Stockholm University, Stockholm, Sweden; 3 Department of Mathematics, Division of Mathematical Statistics, Stockholm University, Stockholm, Sweden

## Abstract

**Background:**

The objective of this study is to analyze the spatial and temporal patterns of malaria incidence as to determine the means by which climatic factors such as temperature, rainfall and humidity affect its distribution in Maputo province, Mozambique.

**Methods:**

This study presents a model of malaria that evolves in space and time in Maputo province-Mozambique, over a ten years period (1999-2008). The model incorporates malaria cases and their relation to environmental variables. Due to incompleteness of climatic data, a multiple imputation technique is employed. Additionally, the whole province is interpolated through a Gaussian process. This method overcomes the misalignment problem of environmental variables (available at meteorological stations - points) and malaria cases (available as aggregates for every district - area). Markov Chain Monte Carlo (MCMC) methods are used to obtain posterior inference and Deviance Information Criteria (DIC) to perform model comparison.

**Results:**

A Bayesian model with interaction terms was found to be the best fitted model. Malaria incidence was associated to humidity and maximum temperature. Malaria risk increased with maximum temperature over 28°C (relative risk (RR) of 0.0060 and 95% Bayesian credible interval (CI) of 0.00033-0.0095) and humidity (relative risk (RR) of 0.00741 and 95% Bayesian CI 0.005141-0.0093). The results would suggest that additional non-climatic factors including socio-economic status, elevation, etc. also influence malaria transmission in Mozambique.

**Conclusions:**

These results demonstrate the potential of climate predictors particularly, humidity and maximum temperature in explaining malaria incidence risk for the studied period in Maputo province. Smoothed maps obtained as monthly average of malaria incidence allowed to visualize months of initial and peak transmission. They also illustrate a variation on malaria incidence risk that might not be related to climatic factors. However, these factors are still determinant for malaria transmission and intensity in the region.

## Background

Malaria is considered one of the most deadly diseases in Mozambique, with around six million cases reported each year [1]. Most of these cases are *Plasmodium falciparum *[1,2]. Transmission takes place all year round with a seasonal peak extending from December to April. Many factors affect the dynamics of malaria transmission and infection, ranging from social to natural. Rainfall and temperature can be considered the major natural risk factors affecting the life cycle and mosquito breeding [2]. Relative humidity plays a role in the lifespan of the mosquito. In the presence of high relative humidity values, the parasite would complete the necessary life cycle in order to increase transmission of the infection to more humans. All districts in Maputo province show favourable climatic conditions for development and transmission of malaria [3]. Studies on prevalence of malaria are important not only to assess the problem of malaria in a given region, but also to analyse the effectiveness of strategies for primary and secondary prevention, as well as its quality and impact.

A combination of advances in hierarchical modelling and geographical information systems has led to the developments in fields of geographical epidemiology and public health surveillance. This made it possible to explore and characterize different sets of spatial disease patterns at a very fine geographical resolution [4]. As a result, disease mapping has been widely used in epidemiology and public health research [5]. The use of geographical mapping helps the detection of areas with high disease incidence for which usually neighbouring areas show similar factors. One common application of disease mapping has been in describing the variation in health outcomes over geographic regions. However, mapping of crude disease rates can be quite misleading particularly at a small area level. This is often due to the combination of two factors: small regional incidence counts and the presence of spatial correlation in the rates. Low prevalence diseases do not provide a possibility of obtaining stable estimates at the district level. For high prevalence diseases like malaria however, these estimates are easily attained due to the availability of a large amount of information at the district level.

Different approaches have been used to model spatio-temporal problems, starting from work by [6] in which space-time interaction is realized by assuming area-specific linear time trend for relative risks. Many other researchers [7,8] proposed and implemented space-time models with different interactions. Spatial and temporal malaria variation is studied in [9] with an investigation of possible geographical expansion of malaria transmission. Space-time models using malaria data are investigated in research by [10,11] where they use dynamic and Bayesian models respectively. Climatic variables are then used as covariate predictors of malaria incidence risk.

This study is motivated by the need to investigate the spatial and temporal variation in malaria rates at the district level in Maputo province - Mozambique, for the period 1999-2008. It is postulated that malaria incidence rates are highly influenced by environmental factors that vary in space and time. To establish this influence, a Bayesian hierarchical model relating malaria incidence rates and climatic data is formulated. The analysis looks at possible relationships between environmental factors and malaria rates to learn about spatial and temporal similarities amongst these rates on different districts of Maputo province. It is expected that the model will facilitate the mapping and elucidation of spatio-temporal patterns of malaria incidence risk. However, before employing environmental factors to explain malaria incidence two issues need to be considered first:

1. The problem of incomplete data, i.e. missing of some explanatory variables. Multiple imputation approach to missing data is pursued.

2. Gelfand *et al *[12] define the change of support problem (COSP) as relating to the inference about the values of a variable measured at different levels of spatial aggregation from those at which it has been observed. In this study, the COSP is addressed by interpolating these factors (covariates) through a Gaussian process.

The aims of this investigation are:

(a) To provide a spatio-temporal analysis of malaria incidence risk;

(b) To determine the contribution of predictors/covariates in the variation of malaria incidence risk

## Methods

### Study area and data

**Figure 1:**
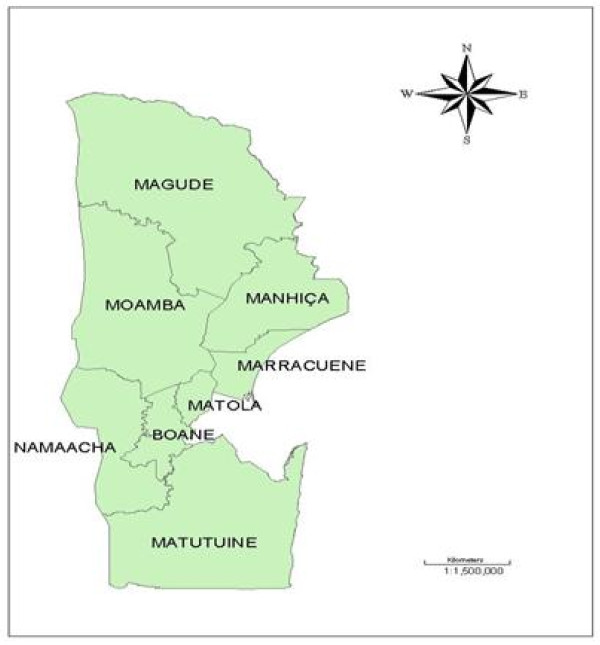
**Study region**.

Given the wide geographic range of Mozambique and weakness of health information system in other regions of the country, a particular region less comprehensive than the whole country was chosen with better quality systems of health recording. Maputo province is located in south of Mozambique with an estimated area of 23,669 square kilometres. Eight administrative districts comprise the province and are shown in the map of Figure 1. Detailed description of study area is given elsewhere [3].

**Figure 2:**
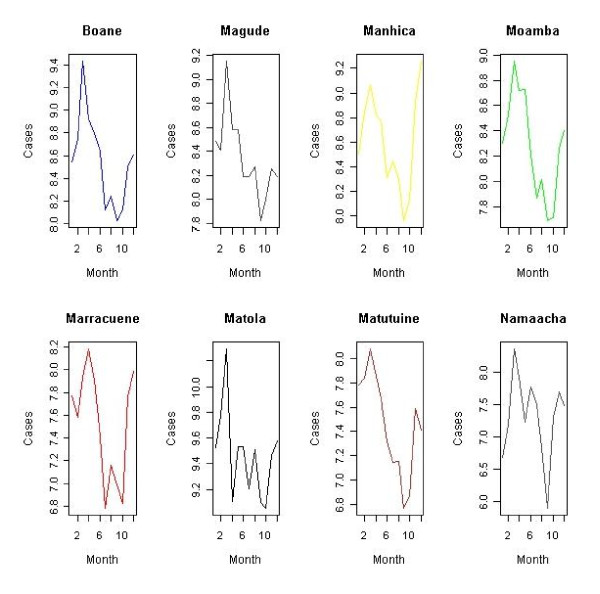
**Variation of monthly malaria cases per district**. Highest malaria trend year 2000.

**Figure 3:**
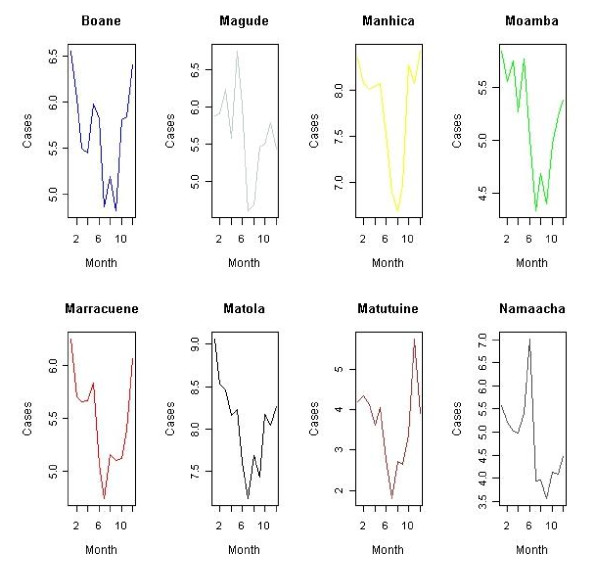
**Variation of monthly malaria cases per district**. Lowest malaria trend, year 2007.

The data comprises malaria rates and environmental averages of rainfall, temperature (minimal, average and maximum) and relative humidity for years 1999-2008 in each month. In fact, the environmental data are available as monthly averages at each monitoring station obtained through the National Institute of Meteorology (INAM), and the response is available as monthly counts of malaria cases in each district. Malaria data includes records from health posts and centres, and rural hospitals compiled at district level in Maputo province [3]. These counts are registered daily and used to generate Weekly Epidemiology Bulletin. It includes both microscopically and clinically confirmed cases. They are collated and summarized by each district health department and reported to provincial health Officers' monthly. The summaries are than sent to the Ministry of Health and shared with different disease control programmes. Expected malaria cases are taken as the number of people in each district according to population projections of 1997 and 2007 national census. Figures 2 and 3, illustrate the variation of malaria cases for years with highest and lowest frequency respectively.

### Modelling

Environmental data used in this study is collected at monitoring stations located in five out of eight districts in Maputo province. This a typical situation of change of support (misaligned) problem with environmental factors observed at fixed locations *s *(point referenced data), whereas the malaria cases are observed at district level. Different approaches are proposed by Zhu and colleagues [12] to tackle the problem of misaligned, namely: predictions from points to points, points to blocks and blocks to blocks. Their work is supported by an application to a static spatial case using the dataset of point-level ozone measurements in the Atlanta metropolitan area. Same researchers in [13] investigated further, by looking at the relationship between ambient ozone and paediatric emergency room visits. The application is extended to spatio-temporal model with log-ozone modelled by a stationary Gaussian process. Before attempting to model misaligned data it was necessary to address the problem of missing data.

#### A. Modelling environmental data

##### A.1 Missing data imputation

In the process of data analysis, it is commonplace to observe that data for each case are not always complete. Rather, some data are usually missing. The amount of missing data may be minimal for some cases; in others perhaps significant. Problems dealing with the analysis of missing data have been extensively reviewed in the scientific literature [14-16]. Environmental time series generally have as their main focus physical and chemical measurements. Hence, problems such as reserved information, privacy respect and non-response as for example in social and medical surveys are not present. The main sources of missing records considered in this study are:

• Break-down of measurement instruments;

• Maintenance interventions, and

• Reading invalidation.

Thus, the data was analysed assuming missing at random mechanism with an incomplete level of environmental observations of around 16.7%. Multiple imputation (MI) package in R [17] was used to perform the imputation of *m = 5 *values for each missing record hence creating five complete datasets. The five imputed values on each variable of interest were aggregated to produce inferential results. The single point estimate was attained by averaging the values of parameter estimates for the covariates rain, humidity and temperature (maximum, mean and minimal values). Table 1 shows the variation and mean of each imputed environmental covariate obtained through MI.

**Table 1:** Overall estimated mean and variance of imputed data.

Estimated	Rain	Max-Temp	Min-Temp	Mean-Temp	Humidity
Mean	73.08	29.44	17.08	23.23	69.16

**Variance**	**3957.74**	**1.861**	**4.934**	**3.21**	**19.586**

In the execution of space-time model with one imputed data set, the results obtained for the main parameters were similar to the run of the model with the data obtained as average values of the five datasets. This assures us that the procedure applied for MI produced consistent results.

##### A.2 Misaligned problem

The change of support problems of interest includes predicting rain, temperature (minimum, mean and maximum) and relative humidity measurements at different points on the map and from that moving to prediction of average weather parameters at district level. Although there are at least twenty-six weather stations registered in Maputo province, on average only five or six stations are operational at any time. The sites are daily monitored within the same time interval and data is aggregated for every four weeks (month). Hence, it comprises time series of spatial processes as the time scale is equally spaced. It is reasonable to consider the change of support problem only in space [13].

Assuming continuous observations from a spatio-temporal process denoted by *Z*(*s*, *t*) at time *t ε T *and location *s ε D*, we seek to change support from observed points to district (block) averages. Following the approach employed in [13], the block average is defined as,(1)

where |B| is the area of block *B *⊂ *D*. Each point in Maputo province is described by its latitude and longitude coordinates through a spatial random field {*Z*(*s*):*s *∈ *D*}, where D ⊂ ℜ*^p ^*and *p *= 2. The amount of rainfall, temperature and humidity *Z_t_*(*B*_1_), ..., *Z_t_*(*B_m_*) ≡ *Z*_1*t*_, ..., *Z_mt _*is predicted for each time *t *(month) in all districts. To obtain predictors for each district (block) *B_i_*, a 90-point grid is overlaid over Maputo province map and the integral in (1) was approximated to a sum. The interpolation is performed by applying Bayesian kriging. Following [4], the random field has to be *Z *= *Z*(*s*_1_), ..., *Z*(*s_n_*) predicted at non-observed point *s*_0_. The model is specified as,(2)

A spatial covariance structure with no nugget effect is used, with Σ specified as Σ = *σ*^2^*H*(*ϕ*) where (*H*(*ϕ*))*_ij _*= *ρ*(*ϕ; d_ij_*). Being the *d_ij _*= || *s_i _*- *s_j _*|| distance between points *s_i _*and *s_j _*and *ρ *is an ordinary exponential function. To complete model specification independent priors are assigned to the parameters namely, a multivariate normal for *β*, inverse gamma for *σ*^2 ^with parameters a = 0.004 and b = 0.02, and a gamma prior for *ϕ *with mean 0.12 and variance about 0.05. The amount of each environmental factor predicted in every district *i *was estimated as the mean of the posterior predictive distribution of the random field process at points of the grid that have fallen within that district. This procedure was independently repeated for every year *r = 1999*, ...*, 2008*.

#### B. Modelling malaria risk in space and time

As a result of preliminary bi-variate analysis performed in statistical package R, the explanatory covariates temperature (minimal, mean and maximum), rainfall and humidity showed a significant relationship p < 0.001 with malaria incidence (Table 2). The data consists of observed malaria counts *O_rit _*and expected cases *E_ri _*in districts *i *= 1*, ..., M *(*M *= 8), for year *r = 1, ..., Years(Years = 10) *and month *t = 1, ..., T(T = 12) *in Maputo province.

**Table 2:** Results of bi-variate analysis of prediction variables.

Covariate	Coefficient	SE	P-value
Min-Temp	.078	.0001	< .001
Mean-Temp	.0694	.0002	< .001
Max-Temp	.0558	.0002	< .001
Rainfall	.001	.000006	< .001
Humidity	.0742	.00014	< .001

Counts of malaria are registered daily at different health centres and rural hospitals generating Weekly Epidemiology Bulletin. They are collated and summarized by each district government health department and reported to provincial health Officers' monthly. These summaries are sent to the Ministry of Health and shared with different disease control programmes. Expected cases are taken as being the population of each district in a corresponding year.

Malaria observed counts are assumed to follow a Poisson distribution with mean *μ_rit_*. The log-relative term modelling all predictor data variables is written as,(3)

where *α *is a measure of overall incidence (intercept term), *θ_i _*is the spatial random effect and *φ_rt _*is the monthly temporal random effect for each year. *δ_rit _*is defined as space and time interaction term with *β *and *X_rit _*being vectors of regression coefficients and environmental covariates respectively. The spatial dependence is introduced through the conditional autoregressive (CAR) process.

In the CAR model, the conditional distribution of each *θ_i _*given all other *θ's *is a normal distribution with mean equal to the average of *θ's *of its neighbours, and precision proportional to the number of neighbours. Hence, a neighbourhood structure needs to be defined and supplied to the model through matrix W. This matrix is important as it specifies how much influence neighbouring districts will have on district *i*. Figure 1, illustrates the location of each district where can be noticed that the shapes and the lengths of their boundaries vary quite a bit among districts.

One simple way to take this information into account is to assign different weights to neighbouring districts according to the length of their boundaries. Therefore, two different specifications of the weighting matrix W are used:

1. Binary structure with *ω_ij _*= 1 for neighbouring districts *i *and *j*, and *ω_ij _*= 0 otherwise;

2. Weighted by the length of the boundary, i.e. with *ω_ij _*equal to the border length (in km) for districts *i *and *j*, and *ω_ij _*= 0 for districts not sharing common boundary. In this case the effect of neighbouring district varies according to the extension of its boundary.

To capture local dependence in time, the year and month temporal trends *φ_rt _*were given a first order random walk prior that allows for year independence. This is a simply one dimensional version of the CAR Normal prior distribution. Hence,(4)

and the weight matrix *Q *defines the temporal neighbours of month *t *as being months *t-1 *and *t+1 *for *t = 2, ..., 11; *with months *t = 1 *and *t = 12 *having singular neighbours.

The space-time interaction terms *δ_rit _*capture departure from space and time main effects which may highlight space-time clusters of malaria risk. In the present study they are assumed to be independent for every year and month with a constant variance over time. This is captured by an auto-regressive AR(1) prior process. It is parameterized by a temporal variance  that allows for correlation between consecutive months within the same district, i.e. assumes that cases at month *t *are influenced by cases of month *t-1*. This relationship holds for months in same year and also for December-January relation of consecutive years, except for January in first and December in last year. A uniform prior was specified to the intercept term and a standard normal prior for coefficients *β *with high variance. Spatial and temporal random effects variance parameters were specified inverse gamma hyper-prior distributions.

**Figure 4:**
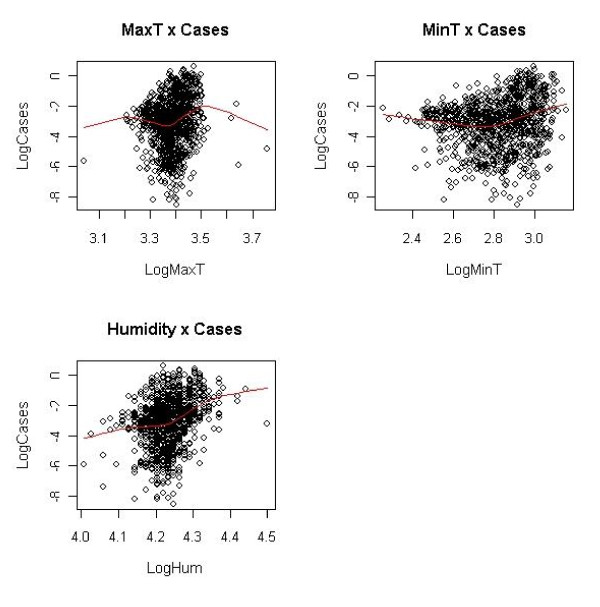
**Illustration of linear relationship of covariates versus malaria cases in log scale**. Piecewise intervals of climatic factors.

The covariate mean temperature was removed from the analysis as it is in general highly correlated with covariate values of maximum and minimum temperatures respectively. However, it was felt necessary to introduce into the analysis the influence of temperature variation on the incidence of malaria, which is modelled by the differences of maximum and minimum temperatures at each time point. Humidity, minimal and maximum temperature showed non-linear relationship to log(*O_rit_*) and were converted to categorical variables for further analysis. Hence, to isolate outliers from the analysis and allow for better observation of the linearity relationship between the variables of the model, the plotting of log-transformed climatic covariates against the ratio of malaria prevalence and population was performed. Their scatter plots are shown in Figure 4. Cut-off points were determined using the statistical package R, with the superimposition of graphs of predictors and the distribution of the ratio of malaria cases and population. For points that did not appear on the × axis, the minimum and/or maximum values (inflection points) were determined, taking also into consideration the change of concavity of the graph. This resulted into having ten coefficients for different threshold intervals with outliers being disregarded.

Model fitting used Markov Chain Monte Carlo (MCMC) simulation techniques implemented in Winbugs with employment of two parallel consecutive chains. A burn-in of 30,000 iterations was allowed where values of main parameters were stored. Diagnostic tests for convergence of stored variables were undertaken, including the analysis of the Brooks, Gelman and Rubin statistics and visual examination of history and density plots, and by computing Monte Carlo errors (MCE). The value MCE/SD was less than 0.05 and thus concluded that sufficient iterations had been conducted. This was followed by a further 30 000 iterations run to obtain posterior distributions of the parameters.

## Results

On average, 324,014 malaria cases were reported per year. Although displaying cases of malaria incidence for just two years, the skewness of malaria data in time series plotted in Figures 2 and 3 is apparently evident. Therefore, there was a need to generate and model smoothed estimates disease risk in order to extract important features from the data. Seven different models were initially analysed following equation (3) and a comparison of their appropriateness performed. This led to dropping of purely temporal structure as being non-important according to its Deviance Information Criteria value [18]. Further refinement considered was the omission of spatio-temporal effect. This resulted into a non-converging spatial model. Table 3 gives the final considered set of models. The DIC analysis shows that model Bwas found to be the best fitted model. Thus, results presented are based on this fitted model.

**Table 3:** Comparison of fitted models.

Model Description	Weight structure	DIC
(A) - Full model	Border lengths	10714.8
(B) - Full model	Binary adjacency	10711.8
(C) - Non-spatial	None	10719.5

Table 4 shows the regression coefficients of the space-time model where it can be seen that rainfall, minimum temperature in the range 11 to 16.4 and maximum temperature in the range 24.5 to 27 degrees centigrade are not associated with incidence risk of malaria in the period under study. However, values of minimum temperature between 17 and 21.1°C, maximum temperature of 28 to 35°C and the occurrence of relative humidity in the range 54.5% to 83% determined positive association with malaria risk. Maximum temperature and humidity in the same range contributed significantly to malaria incidence. Furthermore, an increase of 1°C of maximum temperature leads to higher increase of malaria incidence risk. Whereas, an increase of 1% of relative humidity leads to a variation of malaria from lower to higher incidence risk.

**Table 4:** Posterior estimates of intercept *α*, environmental regression coefficients *β*, of spatial  and spatio-temporal  variances obtained by fitting model B (Table 3), including 95% credible intervals.

Variable	Mean	95% CI	P-value
**Intercept ***(**α**)*	-4.248	-4.389, -4.124	None
**Rainfall (mm)**	-4.30E-04	-9.54E04, 1.01E-05	.059
	**Minimal Temperature (**°**C)**		
**(11-16.4)**	-.005	-.01, 1.11E--04	.055
**(17-21.1)**	2.85E-04	-.003, .005	.96
	**Maximum Temperature (**°**C)**		
**(24.5-27)**	-.001	-.006, .003	.55
**(28-31)**	.002	6.39E-04, .004	.001
**(32-35)**	.006	.003, .009	< .001
	**Relative Humidity (%)**		
**(52-61.56)**	.007	.004, .011	< .001
**(62-72)**	.006	.004, .007	< .001
**(73-83)**	.007	.005, .009	< .001
**Temperature variation**	5.24E-04	-.002 .003	.61
**Spatial variation ()**	3.686	.871, 10.81	None
**Spatio-temporal variation ()**	.2352	.214, .258	None

The effect of humidity and maximum temperature on malaria incidence risk is very high as illustrated by their mean posterior predictive P-value in Table 4. The mean P-value decreases below the critical values as the maximum temperature levels increases. For humidity predictor the mean P-value shows similar pattern. The values estimated by a posterior Bayesian model show a marked variation compared to the Standard Mortality Ratio as shown on maps (See Additional files 1 and 2). There are no similarities in general for regions with both higher SMR and estimated malaria risk. It can be seen that for the months of May to July malaria incidence is very stable. The trends of incidence in the districts of Matutuine and Namaacha are generally lower compared to other districts except for August where this trend increases in the district of Namaacha. For the district of Namaacha, this could particularly be due to being located at higher altitude compared to other districts. Most of its administrative posts lie 400 meters above sea level while the other districts are around 50 meters and surrounded by several water basins. The model using border lengths for weighting matrix did not improve model performance as it is shown by results of DIC analysis in Table 3. It only over-performed the model with no spatial dependency, i.e. non-spatial model.

## Discussion and conclusions

This research have analysed malaria cases data from spatio temporal perspective to identify significant predictors associated with malaria incidence risk and to produce contemporary smoothed maps of disease risk in Maputo province. Maps of smoothed space-time malaria incidence have been produced in several studies [9-11]. Besides the application of techniques of data multiple imputation and spatial alignment in a typical problem analysing the incidence of malaria in Mozambique, this study implements the Bayesian models for analysis with inclusion of temporal random effects and space-time interaction terms.

The problem of missing data is a major issue during the analytical process of any study. This is normally addressed by applying imputation techniques. They follow into two categories: single imputation and multiple imputations (MI). The first has been subjected to increasing criticism by researchers due to its tendency of introducing bias and underestimating standard errors [19]. However, if the quantity of missing values is very small (less than 5%) this methodology can in general be considered accurate. The procedure of multiple imputations is a more general method for inference with missing data. It replaces each missing record with multiple plausible values instead of a single replacement of missing observation.

The mechanism of missing data relates to the underlying reason why the data are actually missing and may follow into three categories [20]:

1. Missing Completely at Random (MCAR): in terms of analysis, no difference established between missing and not missing cases.

2. Missing at Random (MAR): missing data is fully described by variables observed in dataset.

3. Missing Not at Random (MNAR): data missing in an unmeasured fashion termed "non-ignorable"

The establishing of main source of missing records helped to determine and identify the MAR as the most appropriated missing data mechanism underlying the environmental data incompleteness in this study.

The spatial pattern of malaria showed that to the north of Maputo province there is a more pronounced pattern of incidence. In contrast, sub-regions to the centre and south exhibit levels of relatively lower incidence. The main hypothesis for these results could be occurrence of other factors such as indoor pulverization, proximity to water basins, etc. However, the absence of this information has prevented the inclusion of these variables in the analysis. Furthermore, as malaria has a certain period of latency it would ideally be not to include the information about for example present indoor pulverization, but this activity in the past. On the other hand, it is not observed a single temporal gradient of malaria relative risk, with some areas showing a decrease and others exhibit a relative increase. Furthermore, the quantification of relative amount of spatial risk pattern has helped highlighting districts with low and high proportions of malaria incidence at a given time period [11]. In addition, this study may also contribute to the evidence of the importance of spatial and temporal smoothing of random effects in mapping malaria [9,11,21-23].

This study showed that the combination of the monthly maximum temperature in the range 28 to 35°C and relative humidity in the range 54.5% to 83% provided suitable condition for malaria transmission. The negative association attained by maximum temperature in the range of 24 to 27°C to malaria incidence, could indicate a need of warmer temperatures for malaria transmission [11]. The performance of rainfall in the analysis could be influenced by the presence of humidity covariate. High levels of humidity is generally observed when temperature and rainfall are also high, thus leading to suitable conditions of parasite development due to available breeding sites and survival of mosquitoes population [24].

The mapping of averaged smoothed incidence malaria risk for each month and ten years period allows a visually display for months of initial and peak transmission. See Additional files (Additional Files 3 and 4). This may provide information on the length of transmission based on the predicted relationship with the included covariates. Although this study does not present seasonal analysis of malaria incidence variation as in [11], the monthly variation illustrates some seasonal pattern in months May-July (usually considered part of winter period in Mozambique), where the warmer temperatures may have induced the reduction of the die-back mosquitoes and parasite levels, increasing substantially their availability in the following months. Nevertheless, the climate remains the main limiting factor of malaria intensity controlling transmission at both spatial and temporal dimension [25,26].

In conclusion, the models applied in this study adjusted for unobserved spatial and temporal variation on risk factors, while allowing for inter-monthly and inter-annual variation in malaria incidence to be influenced by environmental conditions. Nevertheless, the variation on incidence malaria risks could also be affected by other factors not considered in the analysis. These results may be useful for developing of climate based malaria surveillance systems in Mozambique which can help bring a better management and implementation of nation-wide malaria control programmes, by guiding public and private policies towards reducing malaria incidence in Maputo province. Variation from normal monthly minimal temperature and rainfall patterns in this study, showed their limited use for predicting malaria incidence in Maputo province.

## Abbreviations

MCMC: Markov Chain Monte Carlo; DIC: Deviance Information Criteria; NMCP: National Malaria Control Program; INAM: Mozambique National Meteorology Institute; RR: Relative risk; UEM: Universidade Eduardo Mondlane; SIDA: Swedish International Development Cooperation Agency; CI: Confidence Interval; BES: Weekly Epidemiological Bulletin; CI: Credible Interval; AR(1): First Autoregressive Process; SE: Standard Error.

## Competing interests

The authors declare that they have no competing interests.

## Authors' contributions

MA was responsible for analyses, interpretation of data and results and manuscript's revision. OPZ was responsible for conception and study design, analysis, data interpretation and modelling and manuscript's drafting and revision. The authors read and approved the manuscript.

## Supplementary Material

Additional file 1 **Standard mortality rate maps for months January to July**. Click here for file

Additional file 2 **Standard mortality rate maps for months August to December**. Click here for file

Additional file 3 **Relative risk for months January to June**. Click here for file

Additional file 4 **Relative risk for months July to December**. Click here for file
