# Supplementary figures and images for: Spatial and temporal patterns of malaria incidence in Mozambique
Source: Malar J. 2011 Jul 13;10:189. doi: 10.1186/1475-2875-10-189 (PMC3161914; doi:10.1186/1475-2875-10-189)

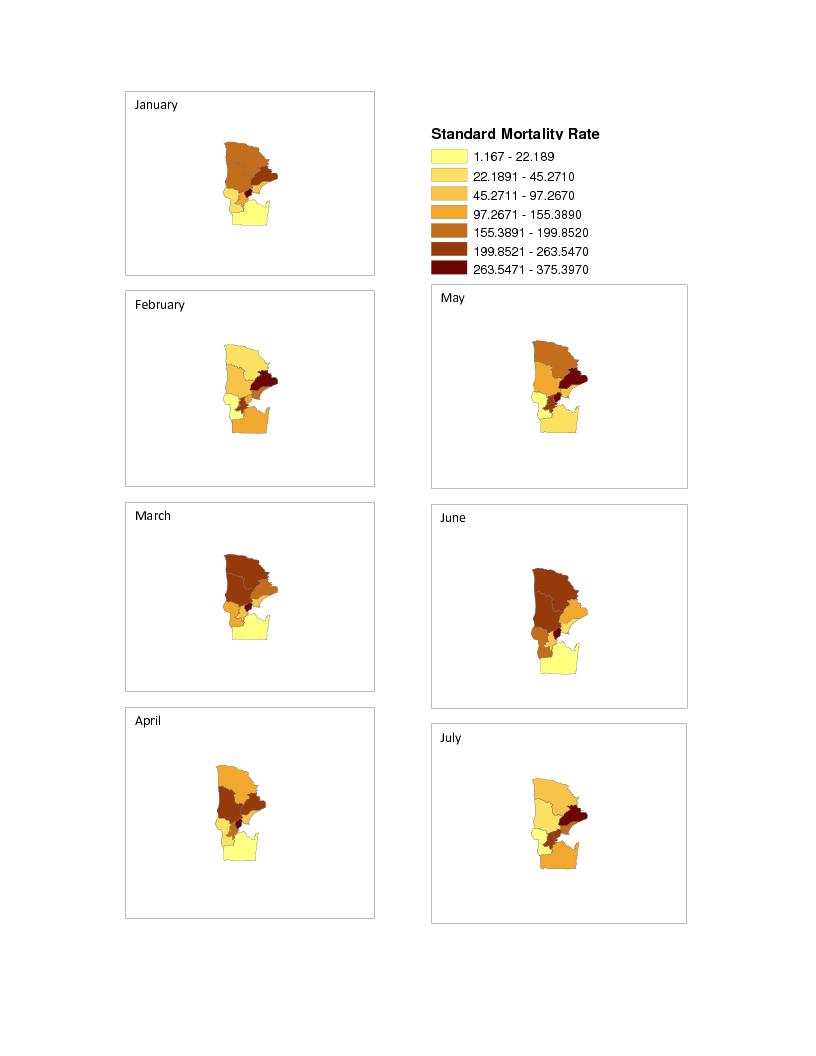

Supplement: Additional file 1 — Standard mortality rate maps for months January to July. [file 1475-2875-10-189-S1.JPEG]

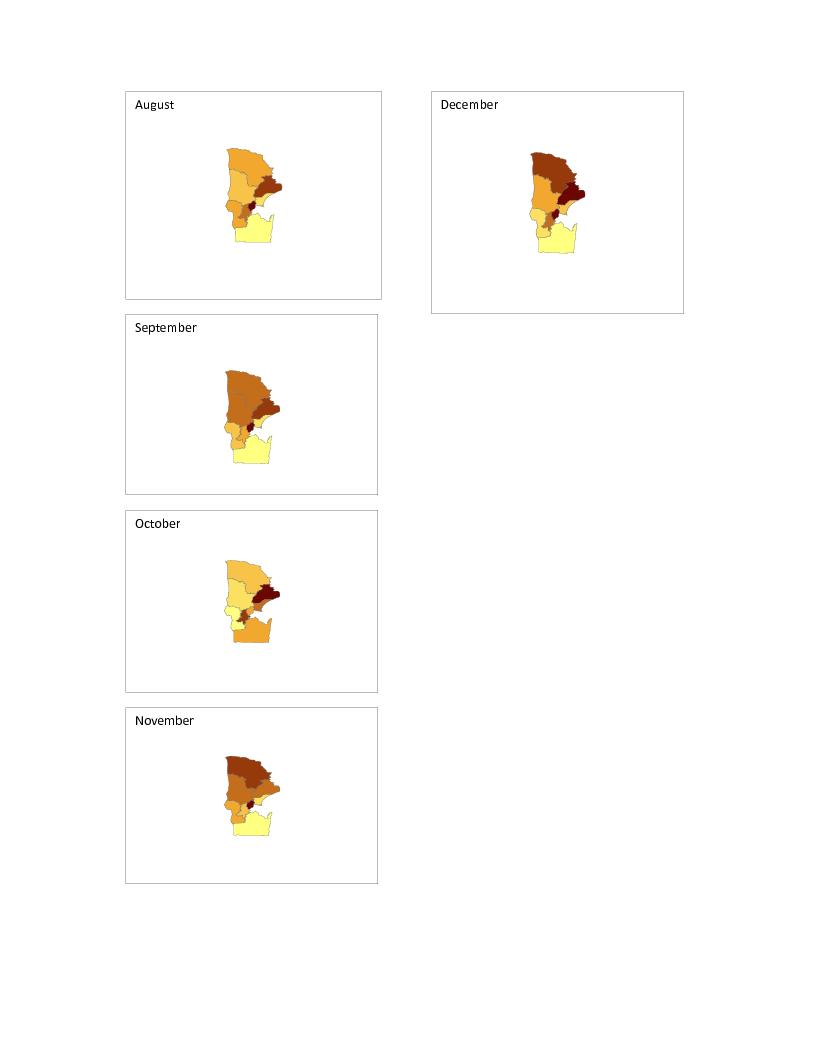

Supplement: Additional file 2 — Standard mortality rate maps for months August to December. [file 1475-2875-10-189-S2.JPEG]

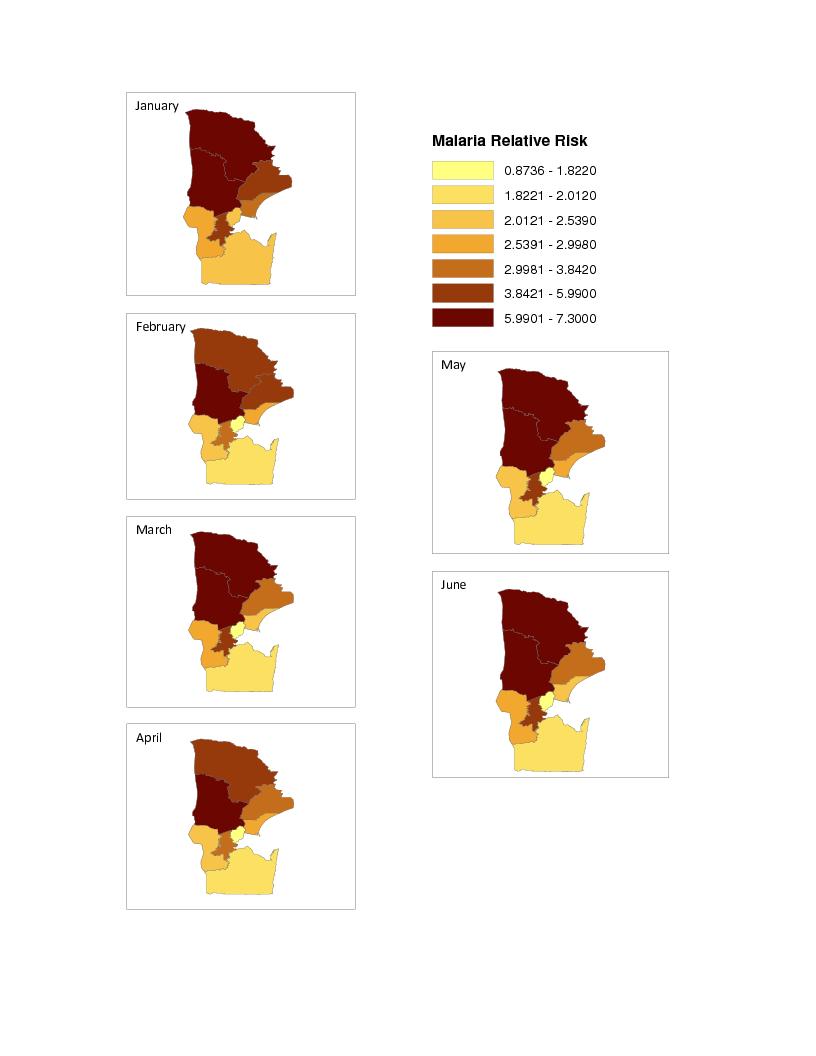

Supplement: Additional file 3 — Relative risk for months January to June. [file 1475-2875-10-189-S3.JPEG]

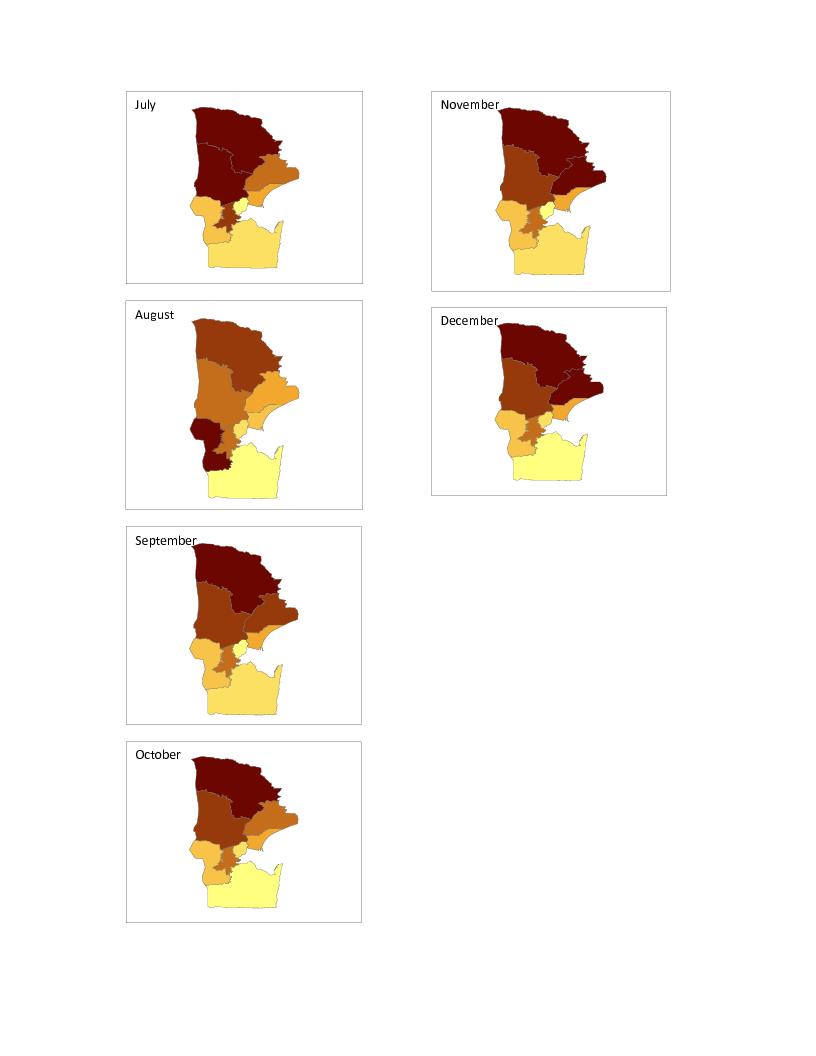

Supplement: Additional file 4 — Relative risk for months July to December. [file 1475-2875-10-189-S4.JPEG]
